# Supplementary material for: Time-Dependent Serial Changes of Antigen-Presenting Cell Subsets in the Ocular Surface Are Distinct between Corneal Sterile Inflammation and Allosensitization in a Murine Model
Source: Cells. 2021 Aug 26;10(9):2210. doi: 10.3390/cells10092210 (PMC8467177; doi:10.3390/cells10092210)
Supplement: Supplementary file 1 [file cells-10-02210-s001.zip › cells-1328775-supplementary/Supplementary Figure S1.pdf]

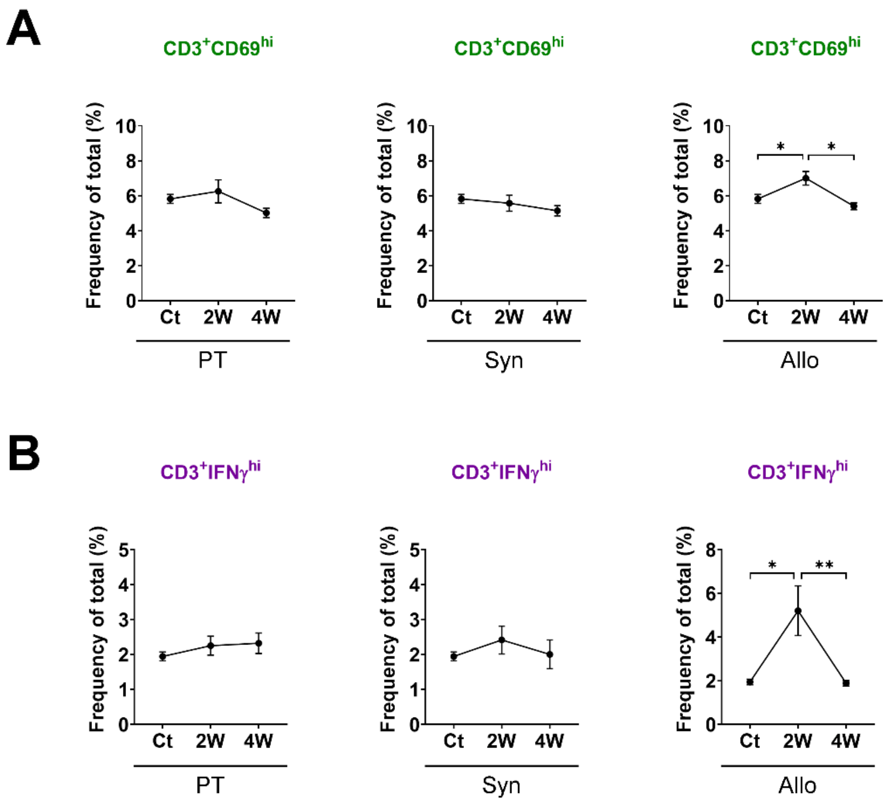

Supplementary Figure S1. Time-dependent serial changes of T cells in draining lymph nodes in response to either sterile inflammation or allosensitization until four weeks.

(**A, B**) Time-dependent frequency (% of total) changes in T cell subsets in the draining lymph nodes. The frequencies (% of total) of the CD69^{hi} and IFNγ^{hi} T cell were increased at two weeks only in the Allo group. n = 5 for each time point and n = 12 for Ct; ANOVA followed by Bonferroni's *post hoc* analyses. *$p < 0.05$ and **$p < 0.01$. Values are expressed as mean ± standard error measurement. Ct indicates the control group; Syn, the syngeneic corneal transplantation group; and Allo, the allogeneic corneal transplantation group. IFNγ, interferon gamma.
