# Supplementary material for: Time-Dependent Serial Changes of Antigen-Presenting Cell Subsets in the Ocular Surface Are Distinct between Corneal Sterile Inflammation and Allosensitization in a Murine Model
Source: Cells. 2021 Aug 26;10(9):2210. doi: 10.3390/cells10092210 (PMC8467177; doi:10.3390/cells10092210)
Supplement: Supplementary file 1 [file cells-10-02210-s001.zip › cells-1328775-supplementary/Supplementary Figure S2.pdf]

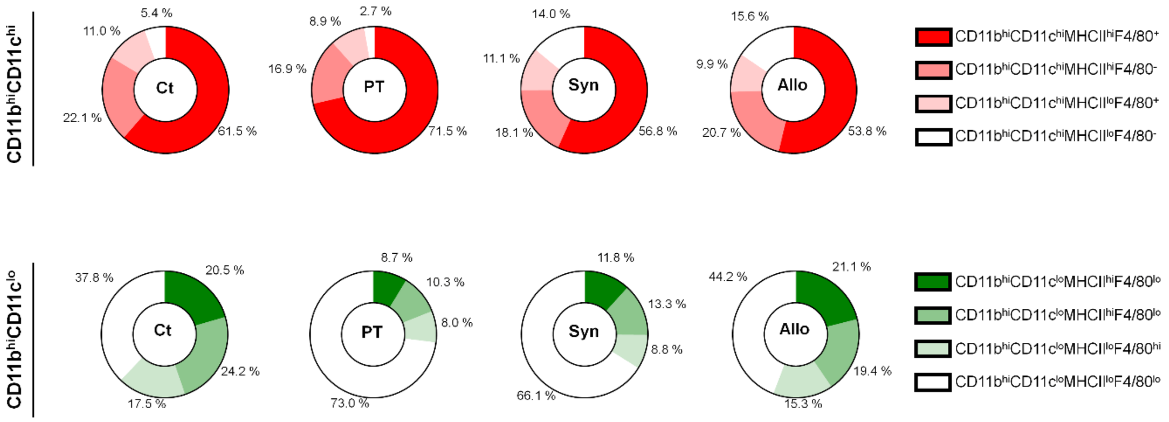

Supplementary Figure S2. Pie charts presenting the distinct distribution patterns of the APC subgroups, depending on the inflammation type in each subset.

The mean percentage of each subset according to the expression of 4 kinds of surface expression markers including CD11b, CD11c, MHCII and F4/80 indicates the fractional proportion within the pool of each CD11b^{hi}CD11c^{hi} and CD11b^{hi}CD11c^{lo} subset. $n = 5$ for each group.
